# Tracheobronchial rhinosporidiosis: an uncommon life‐threatening benign cause of airway obstruction

**DOI:** 10.1002/rcr2.653

**Published:** 2020-08-27

**Authors:** Nirmal Kanti Sarkar, Md. Mofizur Rahman Mia, Md. Rejaul Hasan

**Affiliations:** ^1^ Department of Respiratory Medicine Mugda Medical College Dhaka Bangladesh; ^2^ Department of Thoracic Surgery National Institute of Diseases of the Chest and Hospital Dhaka Bangladesh; ^3^ Department of Anesthesiology National Institute of Diseases of the Chest and Hospital Dhaka Bangladesh

**Keywords:** Bronchoscopy, bronchus, cauterization, rhinosporidiosis, trachea

## Abstract

Rhinosporidiosis is a chronic granulomatous infectious disease caused by Mesomycetozoea *Rhinosporidium seeberi.* This highly recurrent polypoid lesion has a predilection for the nose and nasopharynx, although other organ systems may be affected. Involvement of the tracheobronchial tree is very rare, and poses a challenge for diagnosis and management. In this report, we present a 30‐year‐old man with a history of recurrent nasal polyp who presented with cough, shortness of breath, haemoptysis, and a radiological feature of right lung collapse on imaging. He was diagnosed with rhinosporidiosis based on histopathological examination of bronchoscopic biopsy specimen taken from the right principal bronchial mass. Shortly after hospitalization, he developed acute respiratory distress requiring emergency bronchoscopic intervention. A pinkish mulberry‐like tracheal and right bronchial mass was removed endoscopically with cauterization of the base of the lesion. On long‐term follow‐up, the patient was free of symptoms without recurrence of airway disease.

## Graphical abstract

Tracheobronchial rhinosporidiosis is a rare benign cause of endobronchial obstruction and poses challenge for diagnosis and management. We report a 30‐year‐old man who developed acute hypoxaemia by a rapidly growing rhinosporidial mass causing central airway obstruction. Emergency bronchoscopic intervention saved his life.

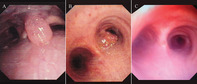

## Introduction

Rhinosporidiosis is a chronic granulomatous infectious disease that manifests as a slow‐growing tumour‐like mass, commonly involving the nose and nasopharynx. The tracheobronchial tree is a very unusual site and poses challenge in management owing to recurrent nature of the disease. Here, we report the case of a patient with a previous history of recurrent nasal mass and multiple nasal surgeries, presented with right upper lobe collapse followed by sudden respiratory distress. Emergency bronchoscopic intervention was life‐saving.

## Case Report

**Figure 1:**
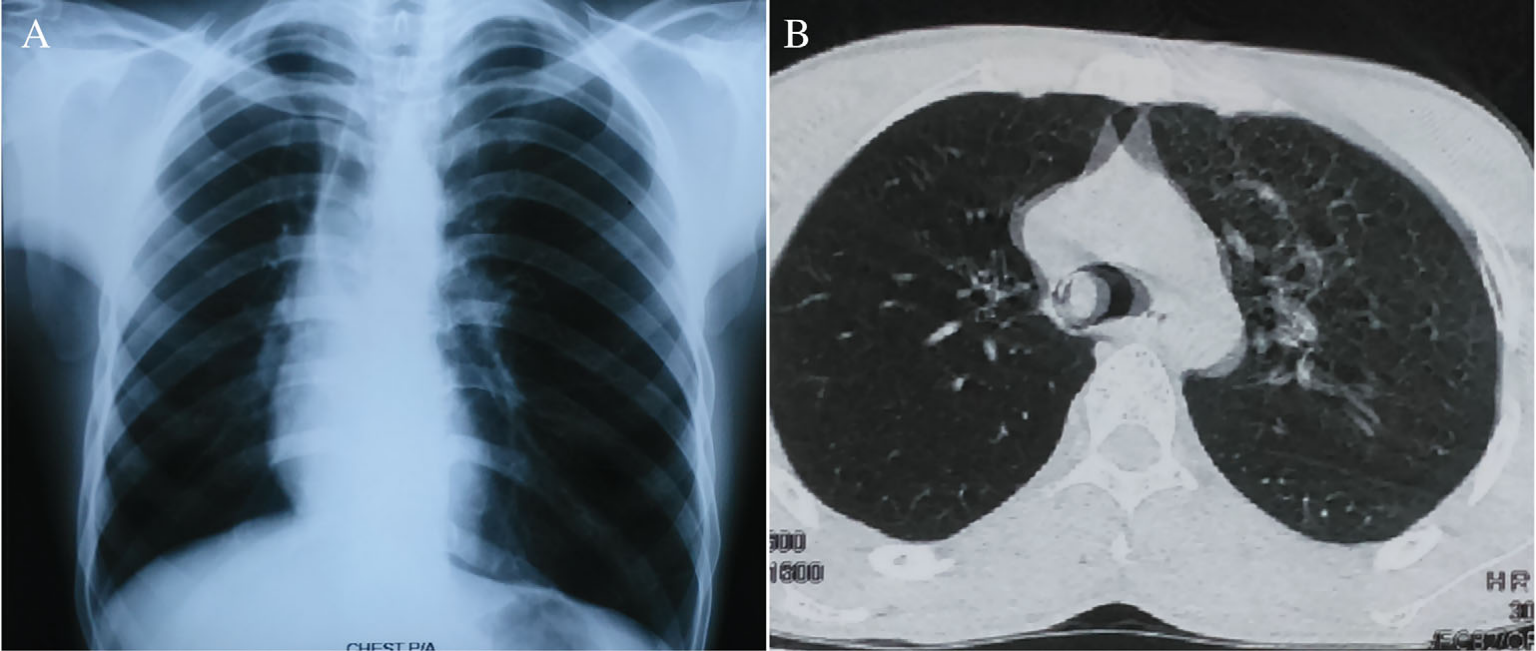
Chest X‐ray P/A view showing the right upper lobe collapse (A), and a polypoid mass at the level of carina on computed tomography scan of the chest (B).

A 30‐year‐old male from a rural background presented to a primary care physician with persistent cough and occasional sputum for six months and was getting treatment accordingly without much improvement. Four months later, he noticed frequent scanty haemoptysis and exertional breathlessness. There was no fever, wheeze, chest pain, and anorexia or weight loss. He was seen by a pulmonologist, and a routine chest X‐ray showed right upper lobe collapse (Fig. 1A). Pulmonary tuberculosis was excluded after obtaining a negative report of sputum for acid‐fast bacilli and GeneXpert (Cepheid, Inc., USA). A right principal bronchial mass was observed on flexible bronchoscopic examination, and histopathology revealed rhinosporidiosis. He was referred to our institute.

**Figure 2:**
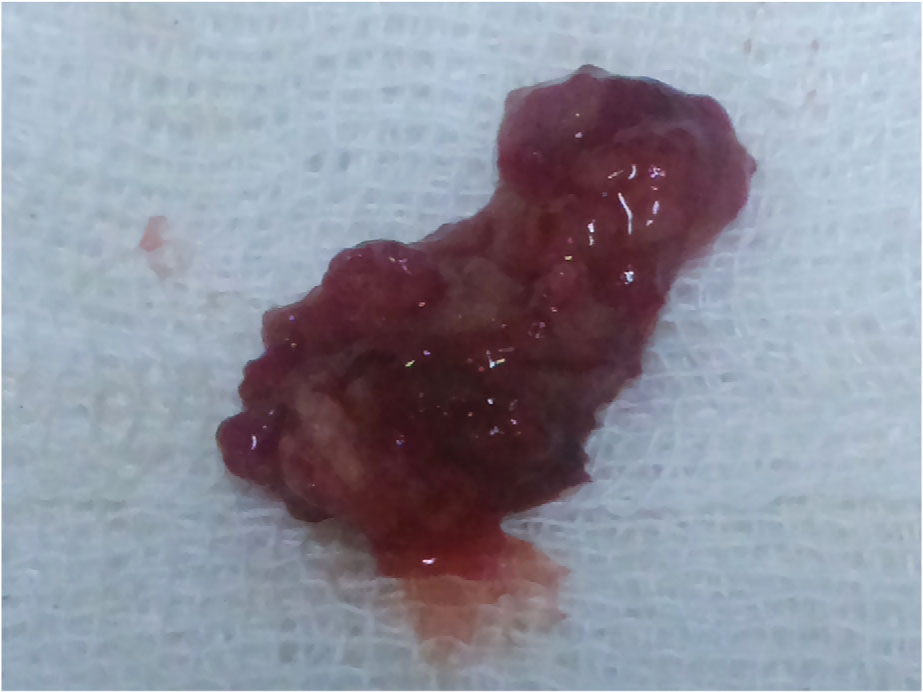
Partially resected rhinosporidial mass.

On admission, he was dyspnoeic with absence of breath sound in the right lung and poor air entry in the left lung. Oxygen saturation (SpO_2_) breathing room air measured 89%. A computed tomography scan of the chest showed a polypoid mass in the right principal bronchus, extending up to the main carina (Fig. 1B). The patient was a farmer who frequently entered local pond water whilst fishing. His history included two previous operations five and eight years prior for management of a nasal mass. However, documentation from these procedures was not available. None of his family members reported such a disease. On the second day of admission, he developed sudden respiratory distress with a fall in SpO_2_ to 72%. Hypoxaemia could not be corrected with 10 L/min supplemental oxygen via a partial rebreathing mask. Emergency rigid bronchoscopy (Karl Storz, Germany) was performed. A mulberry‐like mass was seen nearly occluding the lower tracheal lumen. The mass was removed as much as possible by grasping forceps (Fig. 2), and bleeding was secured by applying pressure with adrenaline‐soaked gauze. The post‐operative period was uneventful. Microscopic examination of the resected specimen showed tissues lined by stratified squamous epithelium. The subepithelial stroma revealed the spherules of *Rhinosporidium seeberi* containing endospores.

**Figure 3:**
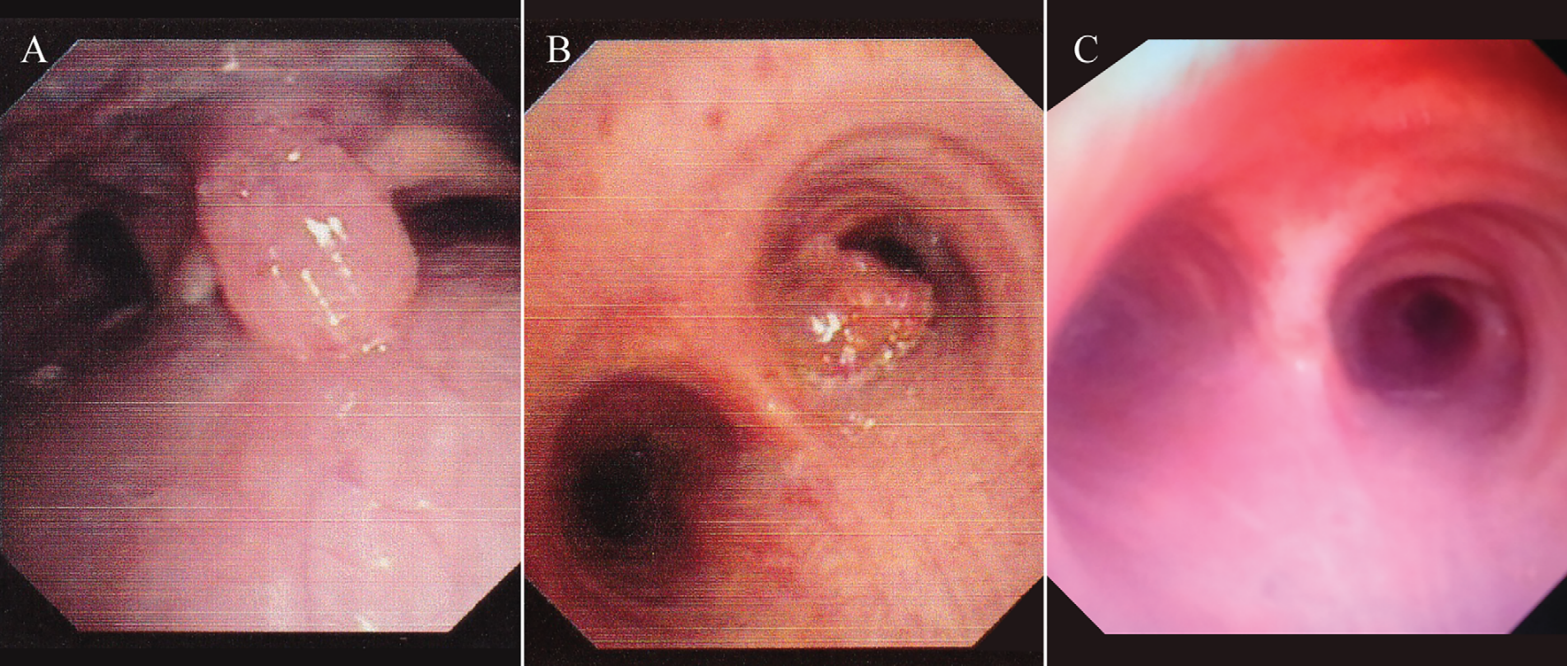
Fibreoptic bronchoscopy one week later showing rhinosporidial mass hanging from the posterior pharyngeal wall (A), and a residual lesion in the right principal bronchus (B). Follow‐up bronchoscopy one and a half months later showing normal tracheobronchial lumen (C).

**Figure 4:**
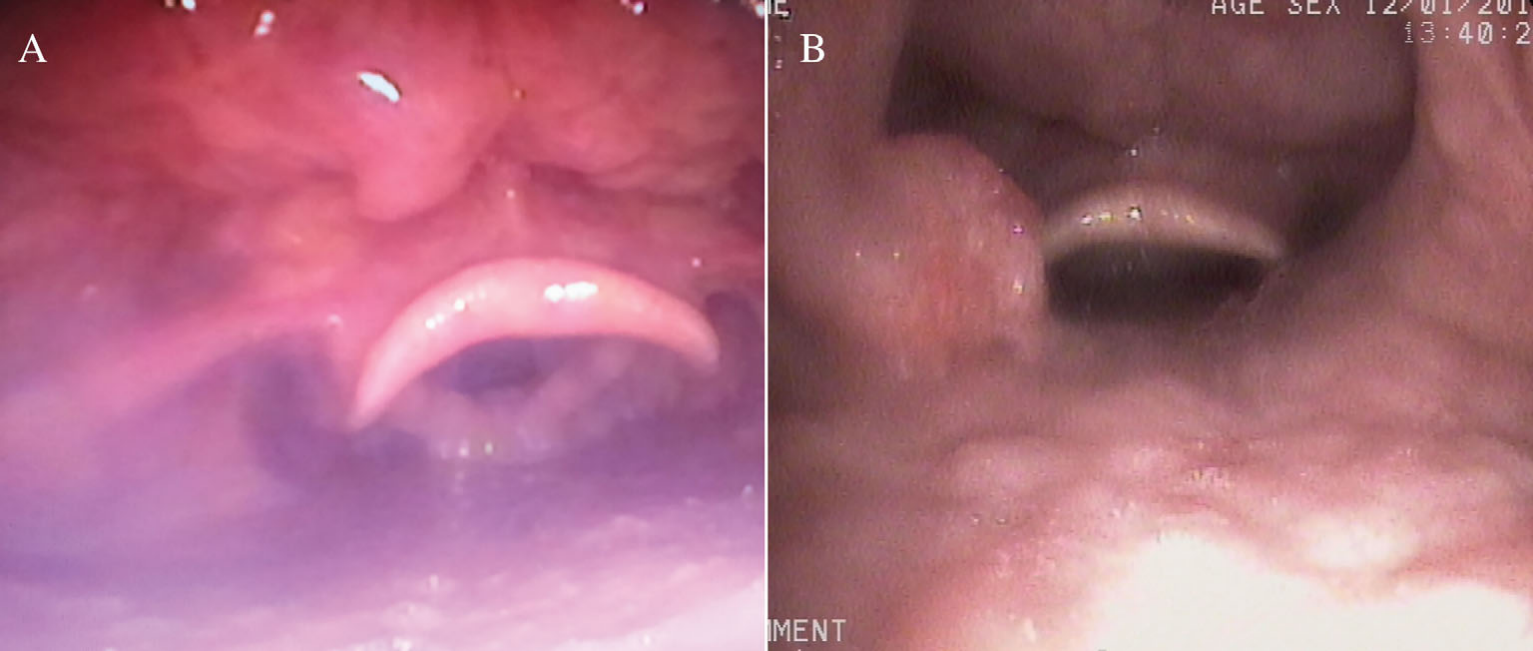
Follow‐up bronchoscopy: one and a half months later—regression of pharyngeal lesion (A), and two years later—recurrence of pharyngeal lesion (B).

One week later, fibreoptic bronchoscopy (FB) showed complete clearance of the tracheal lumen and a residual lesion in the proximal right principal bronchus and a mass hanging from the posterior nasopharyngeal wall (Fig. 3A, B). The rest of the bronchial tree in both lungs was free from any lesion. Rigid bronchoscopy was repeated after seven days to remove the residual lesion of the right bronchus with electrocauterization of the lesional base to prevent recurrence. Dapsone was started at 100 mg/day, and the patient was discharged with advice of monthly follow‐up. Follow‐up bronchoscopy was performed one and a half months later, which showed clearance of the bronchial lesion (Fig. 3C) and regression of the nasopharyngeal mass (Fig. 4A). He continued medication for one year and then stopped by himself. Bronchoscopic evaluation two years later showed airway lumen free of any disease but a recurrent lesion on the left pharyngeal wall (Fig. 4B). Dapsone was started again, and he was referred for otorhinolaryngologist's consultation. At his last visit, he had no respiratory complaint, but there was nasal blockage.

## Discussion

In 1892, Malbran, and later in 1896, Seeber described an apparent sporozoan parasite in nasal polyp in patients living in Buenos Aires, Argentina. In 1923, Ashworth described the life cycle of the organism, argued it as a fungus, and named it *R. seeberi*. Recent phylogenetic analysis suggests that it is a protistan organism in the newly described class Mesomycetozoea of animal–fungus boundary. Its natural host and reservoir are unknown [1, 2]. The disease is reported in the United States, South America, Europe, Africa, and Asia, but most common in the tropics, with highest prevalence in Southern India and Sri Lanka [1]. Although detailed data are not available, the disease is not uncommon in Bangladesh.

*Rhinosporidium seeberi* has an affinity for the mucus membrane of the nasal cavity and nasopharynx. Various other sites may be involved, including the conjunctiva, lacrimal sac, lips, uvula, palate, epiglottis, larynx, maxillary antrum, parotid duct and gland, urethra, penis, vagina, rectum, muscle, skin, and bone [3, 4]. Involvement of the tracheobronchial tree is very rare. The first case of tracheobronchial rhinosporidiosis was reported in 1956 by Thomas et al. [5]. An extensive literature search in PubMed/MEDLINE and Google Scholar using the free text term tracheobronchial rhinosporidiosis revealed only 15 cases, including the index case [3, 5, 6, 7, 8, 9, 10, 11, 12, 13, 14, 15, 16, 17] (Table 1).

**Table 1:** Details of reported cases of tracheobronchial rhinosporidiosis.

	Reference	Age (years)	Gender	Geographical area	Site of airway involved	Other sites of involvement	Treatment	Follow‐up period (months)	Outcome
(1)	Thomas et al. 1956 [5]	31	M	India	RMB, IB	Nose, NPX, lacrimal sac	Lobectomy	31	NR
(2)	Subramanyam et al. 1960 [6]	30	M	India	RMB, trachea	Nose, NPX	—	—	—
(3)	Shah and Ingle 1985 [8]	50	M	India	Lower trachea	Nil	Surgical excision	3	Recurrence
(4)	Puri et al. 2000 [9]	35	M	India	Trachea	Nil	Endoscopic removal, cauterization of base	7	NR
(5)	Rekha et al. 2006 [3]	48	M	India	LMB, trachea	Nil	RB excision, tracheostomy	—	—
(6)	Kini et al. 2010 [10]	30	M	India	RMB, IB	Nose, OPX	Surgical excision	24	NR
(7)	Mathew et al. 2010 [11]	55	M	India	Trachea	Nose, larynx	Radiofrequency ablation via FB	1	NR
(8)	Dhawan et al. 2011 [12]	52	M	India	Trachea	Nil	RB excision	—	—
(9)	Banjara et al. 2012 [7]	35	M	India	LMB	Nose, OPX	FB excision, cauterization of base	12	NR
(10)	Bhate et al. 2012 [13]	41	M	India	Trachea	Nil	Bronchoscopic laser excision	24	NR
(11)	Madana et al. 2013 [14]	45	M	India	Trachea	Nose, NPX, OPX, larynx	RB excision, tracheostomy	24	NR
(12)	Saha et al. 2014 [15]	35	M	Bangladesh	Trachea	Nil	Surgical excision	30	NR
(13)	Hossain et al. 2014 [16]	45	M	Bangladesh	Trachea	Nil	RB excision, tracheostomy	9	NR
(14)	Santhosam 2017 [17]	50	M	India	LMB	Nose, NPX	Surgical excision, cauterization of base	5	NR
(15)	Present case	30	M	Bangladesh	RMB, trachea	NPX	RB excision, cauterization of base	36	NR

FB, fibreoptic bronchoscopy; IB, intermediate bronchus; LMB, left main bronchus; M, male; NPX, nasopharynx; NR, no recurrence; OPX, oropharynx; RB, rigid bronchoscopy; RMB, right main bronchus.

The exact mode of infection is still unknown, but transepithelial transmission has been proposed as a probable mode of infection in natural aquatic environment, and there is a strong relationship between bathing in pond water and getting the disease. In their study, Karthikeyan et al. observed that 59.38% of patients with rhinosporidiosis had a history of bathing in ponds [4]. Organism may be implanted in the lower respiratory tract due to autoinoculation or haematogenous spread [18]. It is obvious that bronchial involvement in our patient was due to the implantation of the parasite from the nose during the previous surgery. The disease appears to be much more common in men than in women from a rural background and low socioeconomic status [4].

Patients may present with cough, wheeze, breathlessness, stridor, haemoptysis, or collapsed lung. If there is critical airway narrowing, there may be rapidly developing respiratory distress and even sudden death [5, 6]. Acute hypoxaemia in our patient was likely due to the rapidly growing bronchial mass extending and occluding the lower trachea, compromising both lungs. Flexible bronchoscopy is a useful tool for the diagnosis, evaluation, and management of tracheobronchial lesions, but caution should be exercised during biopsy, as there is a high risk of bleeding. Computed tomography imaging of the chest and virtual bronchoscopy may provide better details about the extent of the lesion [3].

Various methods have been used in management, including flexible and rigid bronchoscopic snaring, argon plasma coagulation, laser, tracheotomy, tracheostomy, and surgical excision. As our patient developed critical airway obstruction and acute hypoxaemia, we performed emergency rigid bronchoscopy and gently extracted the lesion using forceps, ensuring good haemostasis, and following a certain interval, repeated the procedure and cauterized the base to prevent recurrence. Dapsone (4, 4‐diaminodiphenyl sulphone) was added to treat concomitant nasopharyngeal lesions and to prevent recurrence, which acts by arresting the maturation of sporangia and fibrosis of stroma [19]. The chance of recurrence of nasal rhinosporidiosis is 10%, but that of tracheobronchial lesions is unknown [3].

This challenging case rapidly developed critical central airway obstruction requiring immediate multidisciplinary planning to rescue the airway. Teamwork of interventional pulmonologists, thoracic surgeons, and anaesthesiologists led to a favourable outcome of the uncommon life‐threatening cause of airway obstruction.

### Disclosure Statements

Appropriate written informed consent was obtained for publication of this case report and accompanying images.

This case was presented at the 22nd Congress of the Asian Pacific Society of Respirology.
